# A case of von Hippel–Lindau disease with renal cell carcinoma treated by partial nephrectomy with pre- and post-surgical axitinib therapy

**DOI:** 10.1016/j.eucr.2021.101925

**Published:** 2021-10-30

**Authors:** Takahiro Akioka, Naoki Terada, Hiroki Takamori, Toshio Kamimura, Shoichiro Mukai, Toshiyuki Kamoto

**Affiliations:** Department of Urology, Miyazaki University, Miyazaki, Japan

**Keywords:** Von hippel-lindau disease, Renal cell carcinoma, Partial nephrectomy, Axitinib

## Abstract

von Hippel–Lindau (VHL) disease is an autosomal dominant hereditary disease with benign and malignant tumors occurring in various organs including the kidneys. In patients with renal cell carcinoma (RCC) lesions in both kidneys, it is difficult to determine the treatment strategy. We report a case of VHL disease with RCC treated via partial nephrectomy after 6 months of axitinib therapy. Then, the patient continued to receive low-dose axitinib therapy without any signs of tumor progression for 3 years after surgery. Axitinib combined with surgery might be a treatment option for patients with VHL disease harboring bilateral RCC.

## 1 Introduction

von Hippel–Lindau (VHL) disease is a hereditary disease with autosomal dominant inheritance that presents as cysts, benign tumors, and malignant tumors in various organs. RCC, which is one of the most common causes of death in VHL disease, is found in 24%–45% of patients with VHL disease, and the average age of onset is 39 years.^1^ In patients with multiple RCC lesions, it is difficult to determine the treatment strategy. We usually decide the timing of intervention and select treatment methods such as partial nephrectomy or ablative therapies based on the size and locus of the tumors.

We administered axitinib for 6 months to treat bilateral RCC in a patient with VHL disease, and the tumors dramatically shrank. Then, partial nephrectomy was performed with negative surgical margins followed by additional low-dose axitinib therapy. Residual tumors have not progressed after 3 years without any severe adverse events.

## 2 Case presentation

The case was a 31-year-old man who had not undergone computed tomography (CT) or ultrasound examinations even though his father had VHL disease and died of a brain tumor at 58 years of age. He was aware of a testicular mass, and contrast-enhanced CT revealed multiple testicular cysts, multiple pancreatic cysts, and bilateral renal tumors. In addition, retinal hemangiomas and cerebellar hemangioblastomas were found on magnetic resonance imaging (MRI), and the patient was diagnosed with VHL disease.

**Fig. 1:**
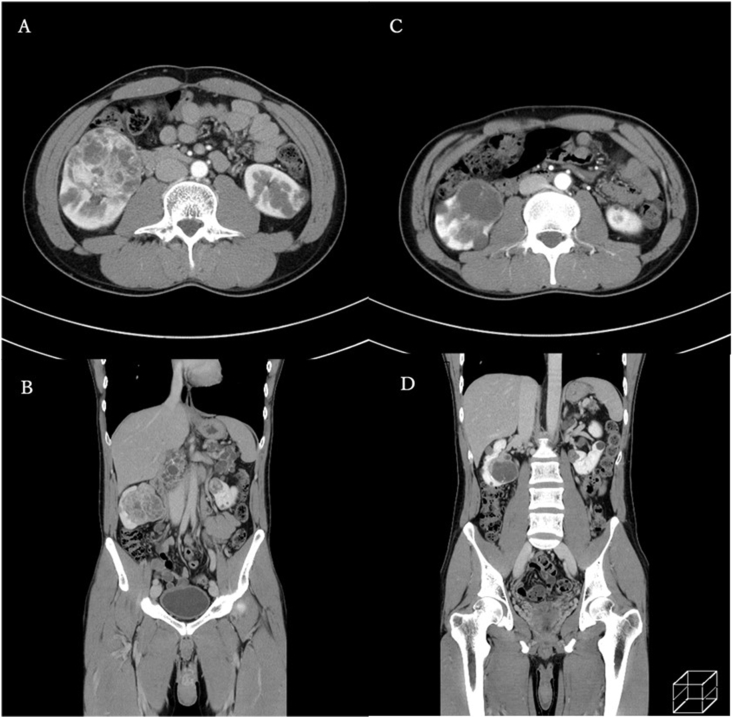
Dynamic computed tomography revealing multiple bilateral renal tumors with increased vascularity (largest diameter, 60 mm) before axitinib therapy (**A:** axial view, **B:** coronal view). Renal tumors exhibited a smaller size and diminished vascularity after 3 months of axitinib therapy (**C:** axial view, **D:** coronal view).

**Fig. 2:**
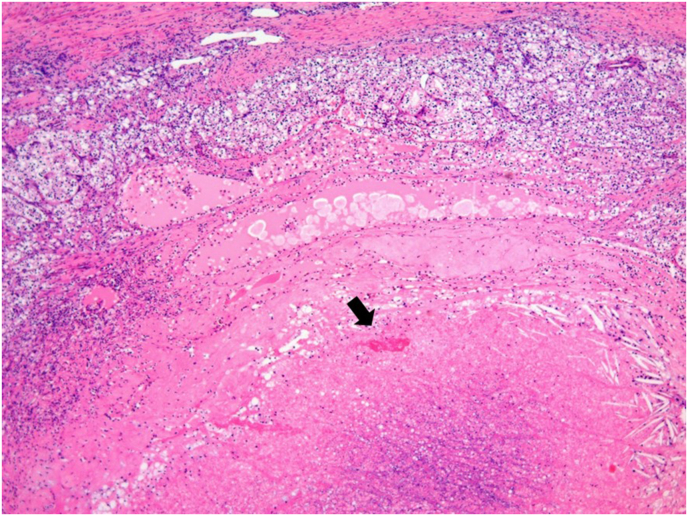
Pathological examination revealing clear cell carcinoma (pT1b, Fuhrman grade 1) with negative surgical margins. Degeneration and necrosis with bleeding were found in the central area (arrow).

Multiple renal tumors with increased vascularity were found in both kidneys. The largest tumor was 60 mm in diameter and located in the right kidney (Fig. 1A and B). He had no venous tumor thrombus, lymph node metastases, or distant metastases. His serum creatinine level was 0.74 mg/dl. The tumor was too large to perform partial nephrectomy, and axitinib administration was started at a dose of 6 mg/day and increased to 10 mg/day after 1 month. Three months later, the largest tumor had shrunk to 48 mm in diameter, and it exhibited diminished vascularity (Fig. 1C and D). The axitinib dose was increased to 14 mg/day, and treatment was continued for an additional 2 months. After 6 months of axitinib treatment, partial right nephrectomy was performed when the tumor diameter was 44 mm. We resected the largest tumor and two additional tumors simultaneously. The pathological diagnosis was clear cell carcinoma, pT1b, Fuhrman grade 1, and negative surgical margins with necrotic tissues indicated the efficacy of axitinib therapy (Fig. 2). The patient's post-operative serum creatinine level was 0.96 mg/dl. He had hematuria in 1 month after the surgery, and transarterial embolization was performed for the pseudoaneurysm.

**Fig. 3:**
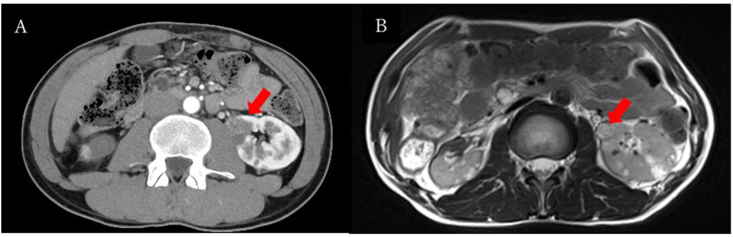
Dynamic computed tomography (**A**) and T2-weighted magnetic resonance imaging (**B**) revealing remaining multiple renal tumors in the left kidney (largest diameter, 10 mm [arrow)] at 3 years after surgery for right renal tumors.

Two months after surgery, several small tumors remained in the left kidney (largest diameter, 10 mm). To prevent tumor growth, 6 mg/day axitinib was started. Six months after surgery, the dose of axitinib was decreased to 4 mg/day, and treatment has continued at this dose. CT and MRI have revealed no signs of tumor growth or metastasis during 3 years of follow-up (Fig. 3). Grade 2 hand-foot syndrome, hypertension, and hypothyroidism as adverse events were controlled by oral calcium channel blockers and levothyroxine.

## 3 Discussion

VHL disease is an autosomal dominantly inherited syndrome that is caused by mutations in the VHL gene located on chromosomes 3p25–26. VHL gene mutation leads to the development of RCC, retinal hemangiomas, central nervous system hemangioblastomas, pancreatic cyst adenomas, pancreatic endocrine tumors, or pheochromocytomas. Because RCC frequently arises in both kidneys, it is necessary to consider the balance between preventing cancer metastasis and avoiding renal failure when determining the treatment strategy. In general, RCC progresses slowly in patients with VHL disease, and <3-cm tumors have a low risk of metastasis.^2^ Therefore, the current standard treatment strategy is surveillance until the tumor size exceeds 3 cm, and nephron-sparing surgery is performed when the tumor size reaches 3–4 cm.^2^ However, repeated partial nephrectomy reduces renal function and increases the risk of complications such as bleeding.^3^ Ablative therapy is also available as an option, and although its short-term results are good, its long-term results are unknown.^2^

Axitinib is a tyrosine kinase inhibitor (TKI) that mainly blocks vascular endothelial growth factor (VEGF). It has several prognostic benefits as a systemic treatment for RCC with or without immune checkpoint inhibitors.^4^ It is considered that the loss of VHL function leads to constitutional activation of the hypoxia-inducible factor (HIF) pathway and the consequent expression of numerous angiogenic and carcinogenic factors including VEGF or other receptor tyrosine kinases.^2^ Ma et al. reported the effect of TKIs on metastatic RCC in 32 patients with VHL, recording partial response and stable disease rates of 28% and 47%, respectively, and median overall survival of 70 months.^5^

In this case, multiple renal tumors existed in both kidneys, and the largest tumor diameter was 60 mm at the time of diagnosis. To safely perform partial nephrectomy, we decided to start pre-surgical treatment with axitinib to achieve tumor shrinkage. Fortunately, the tumors significantly regressed following treatment, illustrating that axitinib was effective in this patient. For residual tumors after the surgery, low-dose axitinib was continued, and tumor progression was prevented for a long period. This case indicated the potential effectiveness of TKIs for multiple RCC lesions in patients with VHL disease considering the mechanisms of RCC development. HIF2α inhibitor belzutifan has been approved by the FDA for patients with VHL-related tumors who require treatment but do not require immediate surgery. However, belzutifan is not available in other countries including Japan. On the other hand, axitinib is globally approved for the treatment of RCC. This case suggests that axitinib could be effectively and safely used instead of belzutifan before it is widely used in the world.

## 4 Conclusion

We reported a case of VHL disease with bilateral RCC in which axitinib shrank the tumors. Partial nephrectomy followed by low-dose axitinib therapy was effective for preventing tumor growth without severe complications for a long period. It was suggested that systemic treatment can potentially prevent renal failure caused by surgical treatment for RCC in patients with VHL disease.

## Declaration of competing interest

None.

## References

[1] Lonser Russel R., Glenn Gladys M., Walther McClellan (2003). von Hippel-Lindau disease. Lancet.

[2] Kim E., Zschiedrich S. (2018). Renal cell carcinoma in von Hippel-Lindau disease―From tumor genetics to novel therapeutic strategies. Front Pediatr.

[3] Johnson A., Sudarshan S., Liu J., Linehan W.N., Pinto P.A., Bratslavsky G. (2008). Feasibility and outcomes of repeat partial nephrectomy. J Urol.

[4] Albiges L., Gizzi M., Carton E., Escudier B. (2015). Axitinib in metastatic renal cell carcinoma. Expert Rev Anticancer Ther.

[5] Ma K., Hong B., Zhou J. (2019). The efficacy and safety of tyrosine kinase inhibitors for Von Hippel-Lindau disease: a retrospective study of 32 patients. Front Oncol.

